# Presepsin as a prognostic biomarker in COVID-19 patients: combining clinical scoring systems and laboratory inflammatory markers for outcome prediction

**DOI:** 10.1186/s12985-024-02367-1

**Published:** 2024-04-26

**Authors:** Zhipeng Wu, Nan Geng, Zhao Liu, Wen Pan, Yueke Zhu, Jing Shan, Hongbo Shi, Ying Han, Yingmin Ma, Bo Liu

**Affiliations:** 1 Department of Respiratory and Critical Care Medicine, Beijing Youan Hospital, Capital Medical University, No. 8, Xi Tou Tiao, Youanmenwai Street, Fengtai District, Beijing City, 100069 People’s Republic of China; 2 Department of Emergency Medicine, Beijing Youan Hospital, Capital Medical University, Beijing City, 100069 People’s Republic of China; 3 Beijing Institute of Hepatology, Beijing Youan Hospital, Capital Medical University, Beijing, 100069 People’s Republic of China; 4 Department of Gastroenterology and Hepatology, Beijing Youan Hospital, Capital Medical University, Beijing, 100069 People’s Republic of China; 5 Beijing Research Center for Respiratory Infectious Diseases, Beijing, People’s Republic of China

**Keywords:** COVID-19, Soluble CD14, Presepsin, Clinical scoring systems, Inflammation-related markers, 28-day mortality

## Abstract

**Background:**

There is still limited research on the prognostic value of Presepsin as a biomarker for predicting the outcome of COVID-19 patients. Additionally, research on the combined predictive value of Presepsin with clinical scoring systems and inflammation markers for disease prognosis is lacking.

**Methods:**

A total of 226 COVID-19 patients admitted to Beijing Youan Hospital’s emergency department from May to November 2022 were screened. Demographic information, laboratory measurements, and blood samples for Presepsin levels were collected upon admission. The predictive value of Presepsin, clinical scoring systems, and inflammation markers for 28-day mortality was analyzed.

**Results:**

A total of 190 patients were analyzed, 83 (43.7%) were mild, 61 (32.1%) were moderate, and 46 (24.2%) were severe/critically ill. 23 (12.1%) patients died within 28 days. The Presepsin levels in severe/critical patients were significantly higher compared to moderate and mild patients (*p* < 0.001). Presepsin showed significant predictive value for 28-day mortality in COVID-19 patients, with an area under the ROC curve of 0.828 (95% CI: 0.737–0.920). Clinical scoring systems and inflammation markers also played a significant role in predicting 28-day outcomes. After Cox regression adjustment, Presepsin, qSOFA, NEWS2, PSI, CURB-65, CRP, NLR, CAR, and LCR were identified as independent predictors of 28-day mortality in COVID-19 patients (all p-values < 0.05). Combining Presepsin with clinical scoring systems and inflammation markers further enhanced the predictive value for patient prognosis.

**Conclusion:**

Presepsin is a favorable indicator for the prognosis of COVID-19 patients, and its combination with clinical scoring systems and inflammation markers improved prognostic assessment.

**Supplementary Information:**

The online version contains supplementary material available at 10.1186/s12985-024-02367-1.

## Introduction

COVID-19 is a systemic disease caused by the novel coronavirus SARS-CoV-2, initially reported in Wuhan, and has had a significant impact on the global socio-economic landscape. Severe patients may experience coagulation dysfunction, acute respiratory distress syndrome (ARDS), and even multi-organ failure [1–3]. With the administration of vaccines and the improvement of the population’s ability to resist the novel coronavirus, the current mortality rate among those infected has significantly decreased [4]. However, due to the continuous mutation of the virus, there are still a significant number of infections, hospitalizations, and deaths occurring [5, 6].

Early prediction of patient prognosis and early intervention can effectively save lives and exploring new prognostic markers is of great significance for the treatment of COVID-19 [7–9]. Inflammatory markers can be used to predict the prognosis of COVID-19 patients [10–12].Early studies have reported the impact of C-reactive protein (CRP), procalcitonin (PCT), erythrocyte sedimentation rate (ESR), serum ferritin, IL-6 on the severity of COVID-19 [13–15]. In addition, various clinical scoring systems such as CURB-65, NEWS2, PSI and others have also been used to predict the prognosis of COVID-19 patients [16–19].

Research has indicated that Presepsin is associated with the prognosis of COVID-19 patients [20, 21]. Presepsin (PSP, sCD14-ST) is a 13 kDa subtype of soluble CD14 (sCD14) that is generated by the cleavage of tissue protease D and other proteases, and it is found in the plasma. CD14 is a member of the Toll-like receptor (TLR) family, capable of binding to lipopolysaccharide (LPS) and promoting innate immune responses. CD14 exists in two forms: membrane-bound CD14 (mCD14) and soluble CD14 (sCD14) [22]. Currently, the biological functions of Presepsin are not fully understood. However, research suggests that Presepsin levels can be considered as an indicator of activation in the innate immune response to pathogen invasion [23]. This marker has been used early on to predict the prognosis of sepsis patients and has shown favorable results [24].

Currently, there is a lack of large-scale studies investigating the value of Presepsin in predicting the prognosis of COVID-19 patients [25]. Furthermore, it is important to note that there is a scarcity of research specifically examining the relationship between Presepsin and COVID-19 prognosis in mainland China. Additionally, there is a lack of research on the combined use of Presepsin with clinical scoring systems and inflammation-related markers for predicting the prognosis of COVID-19.

To address this question, we evaluated the predictive value of Presepsin and compared it with other markers such as C-reactive protein (CRP), procalcitonin (PCT), and blood count-derived inflammatory markers (BCDIMs) including the neutrophil-to-lymphocyte ratio (NLR), monocyte-to-lymphocyte ratio (MLR), platelet-to-lymphocyte ratio (PLR), and others. Additionally, we assessed the predictive value of clinical scoring systems such as SOFA, CURB-65, COVID-GRAM, and NEWS2 for the prognosis of COVID-19 patients. Finally, we evaluated the value of combining Presepsin with clinical scoring systems and inflammatory markers in predicting the prognosis of COVID-19 patients.

## Patients and methods

### Study design and participants

We conducted a retrospective study involving 226 COVID-19 patients admitted to Beijing You’an Hospital’s emergency department between May 1st and November 30th, 2022. All patients were diagnosed according to the recommendations of the World Health Organization. Based on the guidelines from the National Health Commission of China’s “Diagnosis and Treatment protocol for COVID-19 patients (tentative 9 version).“ [26], patients were classified as having mild/moderate or severe/critical COVID-19 cases. This study aimed to evaluate the predictive value of Presepsin, clinical scoring systems, and inflammatory markers on the severity and prognosis of COVID-19 patients at the time of admission. The primary outcome was the 28-day mortality rate. The study was approved by the Ethics Committee of Beijing You’an Hospital, Capital Medical University, and adhered to the principles of the Helsinki Declaration (Approval No. LL-2023-006-K). All participating patients provided informed consent, and the data used in the study were anonymized.

### Inclusion and exclusion criteria

Inclusion Criteria: (i) Patients who met the Diagnosis and Treatment protocol for COVID-19 patients (tentative 9th version) released by the National Health Commission of China [26]. (ii) Patients who agreed to participate in the study and provided informed consent. Exclusion Criteria: i) Patients who did not agree to participate in the study.

ii) Patients below the age of 18. iii) Patients who died within 48 h of hospital admission. iv) Pregnant women. v) Patients with unavailable or lost follow-up blood samples.

### Data collection

The demographic data, comorbidities, baseline characteristics, vital signs, arterial blood gas, laboratory data, and prognosis status were extracted from electronic medical records. The severity of illness categories were defined according to the COVID-19 treatment guidelines recommended by the National Institute of Health. These categories include asymptomatic infection, mild illness, moderate illness, severe illness and critical illness, based on a range of clinical manifestations (source: https://www.covid19treatmentguidelines.nih.gov/overview/clinical-spectrum/). The comorbidities considered in the study were hypertension, diabetes mellitus (DM), coronary heart disease, malignancies, cerebrovascular disease, chronic obstructive pulmonary disease (COPD), liver disease, kidney disease, and malignant tumor.

The vital signs assessed in the study included body temperature measured in degrees Celsius (℃), respiratory rate (RR) measured in breaths per minute, heart rate (HR) measured in beats per minute, and systolic blood pressure (SBP) measured in millimeters of mercury (mmHg). The laboratory parameters included in the study were complete blood count (CBC) which consists of hemoglobin (HGB) level, white blood cell (WBC) count, neutrophil count, and lymphocyte count. Additionally, biochemical function tests were conducted, measuring procalcitonin (PCT) levels in ng/mL, C-reactive protein (CRP) levels in mg/L, international normalized ratio (INR), D-dimer levels in mg/L, glucose levels in mmol/L, alanine transaminase (ALT) levels in U/L, aspartate transaminase (AST) levels in U/L, total bilirubin (TBIL) levels in µmol/L, and direct bilirubin (DBIL) levels in µmol/L. As shown in Table 1.

**Table 1:** Baseline characteristics and clinical data after hospitalization of study population

Variables	Total (*n* = 190)	28-day survival (*n* = 167)	28-day mortality (*n* = 23)	P-value
**Demographic data**				
Sex, male, n (%)	111 (58%)	99 (59%)	12 (52%)	0.517
Age (years)	69 (59–78)	69 (57–77)	82 (69–88)	0.001*
**Co-morbidities**				
Hypertension, n (%)	80 (42%)	67 (40%)	13 (57%)	0.135
Diabetes mellitus, n (%)	42 (22%)	33 (20%)	9 (39%)	0.036*
Coronary heart disease, n (%)	35 (18%)	31(19%)	4(17%)	0.892
Cerebrovascular disease, n (%)	29 (15%)	21(13%)	8 (35%)	0.005*
COPD, n (%)	22 (12%)	20 (12%)	2 (9%)	0.638
Liver disease, n (%)	29 (15%)	25 (15%)	4 (17%)	0.762
Kidney disease, n (%)	26 (14%)	20 (12%)	6 (26%)	0.065
Malignant tumor, n (%)	26 (14%)	22 (13%)	4 (17%)	0.581
**Vital signs**				
Body temperature, ℃	36.6 (36.2–37.0)	36.6 (36.2–37.0)	37.5 (37.0–38.0)	< 0.001*
RR, breaths/min	20 (20–23)	20 (20–22)	25 (22–30)	< 0.001*
HR, beats/min	87 (78–99)	86 (78–98)	98 (83–104)	0.035*
SBP, mmHg	130 (119–144)	130 (120–144)	127 (114–137)	0.219
**Arterial blood gas**				
PH	7.418 (7.395–7.441)	7.419 (7.397–7.440)	7.410(7.370–7.463)	0.924
PaCO_2_, mmHg	37.9 (34.0-40.5)	37.9 (34.5–40.7)	37.4 (27.1–39.9)	0.200
PaO_2_, mmHg	96.0 (76.7–110.0)	96.3 (80.5–110.0)	75.2 (58.5-108.3)	0.029*
SpO_2_, %	98.2 (96.3–99.0)	98.3 (96.9–99.0)	95.6 (92.9–99.3)	0.027*
PaO_2_/FiO_2_, mmHg	282 (207–330)	290 (232–333)	115 (70–215)	< 0.001*
**COVID-19 severity class, n (%)**				< 0.001*
Mild illness	83 (44%)	81 (49%)	2 (9%)	
Moderate illness	61 (32%)	61 (37%)	0 (0)	
Severe/critical illness	46 (24%)	25 (15%)	21 (91%)	
**Clinical scoring system**				
sSOFA	1 (1–2)	1 (1–2)	1 (1–2)	0.031*
eSOFA	0 (0–1)	0 (0–1)	1 (1–3)	< 0.001*
qSOFA	0 (0–1)	0 (0–1)	2 (2–2)	< 0.001*
SOFA	3 (2–4)	2 (2–3)	5 (4–6)	< 0.001*
NEWS2	4 (2–7)	3 (2–5)	9 (8–11)	< 0.001*
PSI risk class	3 (2–4)	3 (2–4)	5 (4–5)	< 0.001*
PSI	91(38)	84 (30)	147(43)	< 0.001*
COVID-GRAM	128 (99–150)	122 (95–143)	171 (157–193)	< 0.001*
CURB-65	1 (0–2)	1 (0–1)	3 (3–4)	< 0.001*
**Laboratory parameters**				
Presepsin, pg/mL	275 (169–536)	245 (165–437)	817 (689–1117)	< 0.001*
PCT, ng/mL	0.07 (0.05–0.17)	0.07 (0.05–0.13)	0.34 (0.12–0.88)	< 0.001*
CRP, mg/L	18.85 (4.90–51.90)	15.5 (4.5–45.8)	68.3 (36.2-103.2)	< 0.001*
HGB, g/L	125 (109–138)	126 (110–138)	123 (104–130)	0.246
WBC count, ×10^9^/L	4.85 (3.77–6.58)	4.78 (3.77–6.46)	5.66 (4.60–7.20)	0.153
Neutrophils count, ×10^9^/L	3.37 (2.27–5.09)	3.32 (2.25–4.81)	4.73 (2.80–6.83)	0.075
Lymphocytes count, ×10^9^/L	0.93 (0.66–1.30)	0.97 (0.72–1.34)	0.66 (0.39–0.94)	0.004*
INR	1.05 (1.01–1.14)	1.05 (1.01–1.13)	1.08 (1.01–1.22)	0.315
D-dimer, mg/L	203 (9-471)	193 (10–377)	464 (1.3–985)	0.074
Glucose, mmol/L	6.7 (6.0-7.8)	6.7 (6.0-7.6)	7.4 (6.4–10.5)	0.030*
ALT, U/L	19 (13–28)	19 (13–28)	23 (18–27)	0.311
AST, U/L	24 (18–33)	22 (17–30)	37 (22–55)	< 0.001*
TBIL, µ mol/L	10 (8–15)	10 (8–15)	11 (10–17)	0.111
DBIL, µ mol/L	3.9 (2.6–6.5)	3.7 (2.5–6.2)	6.2 (4.2–7.7)	0.006*
CAR	0.538 (0.136–1.636)	0.398 (0.125–1.266)	2.020 (1.175–3.360)	< 0.001*
**BCDIMs**				
NLR	3.54 (2.09–6.74)	3.41 (2.08–5.98)	5.92 (4.02–9.19)	0.006*
MLR	0.40 (0.26–0.70)	0.40 (0.27–0.65)	0.52 (0.33–0.86)	0.064
PLR	159 (116–231)	152 (115–224)	182 (156–323)	0.083
LCR	0.046 (0.015–0.197)	0.066 (0.020–0.213)	0.011 (0.005–0.018)	< 0.001*
SIRI	1.306 (0.668–3.240)	1.245 (0.663–2.929)	2.661 (1.221–5.156)	0.048*
SII	490 (296–1022)	476 (288–905)	1022 (480–1242)	0.025*

Normally distributed continuous variables are displayed as mean ± standard deviation (SD) and were compared using the independent-samples Student’s *t test*. Non-normally distributed continuous variables are displayed as a median with interquartile range (IQR: Q1-Q3) and were compared using the Mann–Whitney U test. Categorical variables are expressed as counts with percentages and were compared using Pearson’s chi-square or Fisher’s exact test. *Abbreviations* COPD, Chronic Obstructive Pulmonary Disease; RR, respiratory rate; HR, heart rate; SBP, systolic blood pressure; PaCO_2_, arterial carbon dioxide tension; PaO_2_, oxygen tension; SpO_2_, peripheral oxygen saturation; FiO_2_, fraction of inspired oxygen; sSOFA, simplified sequential organ failure assessment; eSOFA, early sequential organ failure assessment; qSOFA, quick sequential organ failure assessment; SOFA, sequential organ failure assessment; NEWS2, National Early Warning Score 2; PSI, Pneumonia Severity Index; PCT, Procalcitonin; CRP, C-reactive protein; HGB, Hemoglobin; WBC, White blood cell; INR, International normalized ratio; ALT, Alanine aminotransferase; AST, Aspartate aminotransferase; TBIL, Total bilirubin; DBIL, Direct bilirubin; BCDIMs, blood count-derived inflammatory markers; NLR, Neutrophil-to-lymphocyte ratio; MLR, Monocyte-to-lymphocyte ratio; PLR, Platelet-to-lymphocyte ratio; LCR, Lymphocyte-to-C-reactive protein ratio; CAR, C-reactive protein-to-albumin ratio; SIRI, Systemic inflammation response index; SII: Systemic inflammation index. SIRI = (Neutrophil count × Monocyte count) / Lymphocyte count; SII = (Neutrophil count × Platelet count) / Lymphocyte count

*p-value < 0.05 was considered significant

**Fig. 1:**
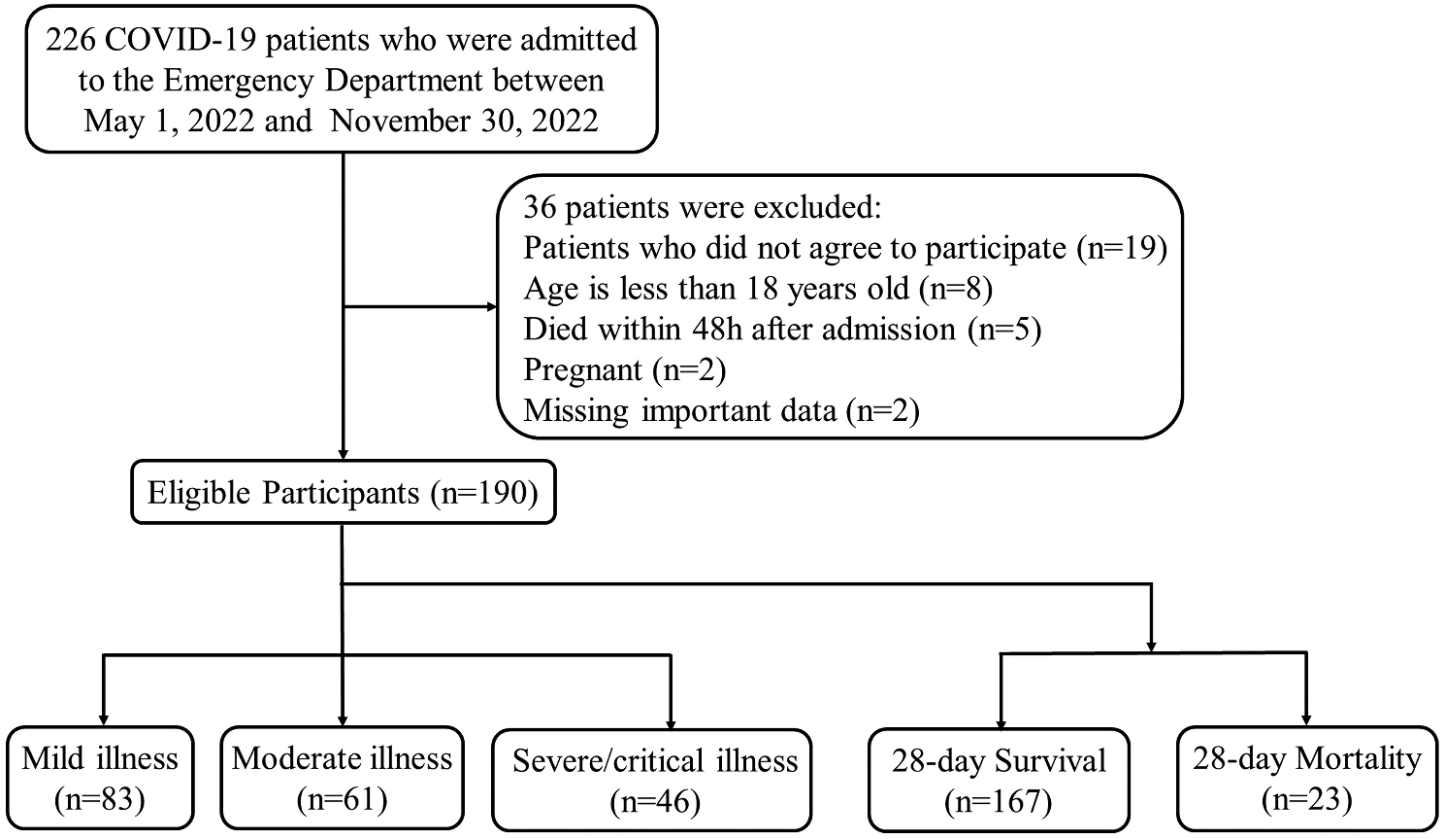
Flow diagram of patients enrollment

Since the number of patients included in our study was not very large, and the collected data mainly consisted of routine clinical indicators, there were only a few patients with missing data. For those patients with missing data, we chose to exclude them from the analysis (Fig. 1). Our analysis is based on the raw data, without further normalization or encoding of nominal data.

### Blood sample collection and testing

Blood sample collection and testing were performed simultaneously during the first blood draw upon admission. The blood samples were collected in anticoagulant tubes containing ethylenediaminetetraacetic acid (EDTA). The plasma was obtained by centrifuging the whole blood at 1350 g for 12 min and then stored at -80 °C for further measurement of Presepsin levels. The concentration of plasma Presepsin was determined using a compact automated immunoassay analyzer based on chemiluminescent enzyme immunoassay (PATHFAST; Mitsubishi Chemical Medicine Co., Ltd., Tokyo, Japan).

### The definition of clinical scoring systems and derived scores for inflammatory markers

SOFA (Sequential Organ Failure Assessment), qSOFA (quick SOFA), eSOFA (early SOFA), and sSOFA (simplified SOFA) are scoring systems used to assess the degree of organ dysfunction in critically ill patients. A useful diagnostic tool for predicting hospital mortality in critically ill adult patients with suspected infections in the intensive care unit (ICU) [27–29].

The CURB-65 score is a well-established severity scoring system used to assess the severity of pneumonia. It includes five criteria: confusion of mental status, urea level, respiratory rate, blood pressure, and age equal to or greater than 65 years [17, 30].

NEWS2 (National Early Warning Score 2) is a scoring system used to assess the clinical condition and detect early signs of deterioration in patients. It consists of the following parameters: respiratory rate, SpO_2_ (oxygen saturation), supplemental oxygen use, heart rate, altered consciousness, and temperature [18, 19].

The Pneumonia Severity Index (PSI) includes 20 independent risk factors and is widely used for assessing the severity of pneumonia. Based on the score, it categorizes patients into five risk classes or levels [31, 32].

COVID-GRAM was first proposed by Chinese scholars in 2020. It includes 10 independent predictive factors and is used for early prediction of the progression to severe illness in COVID-19 patients [33].

Derived scores for inflammatory markers are calculated by combining two or more laboratory parameters, including: NLR, Neutrophil-to-lymphocyte ratio; MLR, Monocyte-to-lymphocyte ratio; PLR, Platelet-to-lymphocyte ratio; LCR, Lymphocyte-to-C-reactive protein ratio; CAR, C-reactive protein-to-albumin ratio; SIRI, Systemic inflammation response index; SII: Systemic inflammation index. SIRI = (Neutrophil count × Monocyte count) / Lymphocyte count; SII = (Neutrophil count × Platelet count) / Lymphocyte count.

### Statistical analysis

The normality of continuous variables was assessed using the Shapiro-Wilk test. Normally distributed continuous variables are presented as mean ± standard deviation (SD) and were compared using independent-samples Student’s t-test. Non-normally distributed continuous variables are presented as median with interquartile range (IQR: Q1-Q3) and were compared using the Mann-Whitney U test. Categorical variables are reported as counts with percentages and were compared using Pearson’s chi-square or Fisher’s exact test. Multiple samples were compared using the non-parametric Kruskal-Wallis test, for example, comparing the levels of Presepsin among four groups of patients: Mild, Moderate, Severe, and Critical. Variables with P values < 0.05 were considered statistically significant. The Receiver Operating Characteristic (ROC) curve is used to assess the predictive performance of parameters for 28-day mortality in COVID-19 patients. Spearman’s rank correlation is used to analyze the correlation between Presepsin and age, clinical score systems, and laboratory markers of inflammation. The least absolute shrinkage and selection operator (LASSO) binary logistic regression model is employed to select clinical score systems and inflammation-related factors. The optimal parameter (λ) is selected using 10-fold cross-validation based on the criteria of 1 standard error of the minimum. Multivariable Cox regression analysis and Kaplan-Meier curves are utilized to evaluate the risk prediction of parameters for 28-day mortality in COVID-19 patients.

Nomograms are developed by combining Presepsin with clinical scoring systems and inflammation-related factors. The calibration of the nomograms is evaluated using the Hosmer-Lemeshow goodness-of-fit test and calibration curves. The clinical utility of the models is also assessed through decision curve analysis (DCA). Data were analyzed using SPSS software (version 22.0; IBM Corp.) and R language (version 4.2.1; R Foundation for Statistical Computing) and illustrated using GraphPad Prism 9 (GraphPad Software Inc.).

## Results

### Patient characteristics and clinical parameters upon admission

Among the 226 patients admitted to the emergency department, 190 patients met the criteria for further analysis. The patient enrollment process is shown in Fig. 1. Among them, there were 83 cases (43.7%) classified as mild, 61 cases (32.1%) as moderate, and 46 cases (24.2%) as severe or critically ill. Ultimately, 23 cases (12.1%) died within 28 days of admission.

Table 1 describes the baseline characteristics and clinical parameters of the patients. Of the patients, 111 (58%) were male, and the median age was 69 years. The most common comorbidities among the patients were hypertension (80/190, 42%) and diabetes (42/190, 22%).

Compared to the patients who survived for 28 days, those who died within 28 days were older, had a higher proportion of diabetes and cerebrovascular disease, had higher body temperature upon admission, faster respiratory and heart rates, lower oxygen partial pressure and oxygenation index, and higher clinical scoring system scores (all p-values < 0.05). In terms of laboratory parameters, the 28-day mortality group had higher levels of Presepsin, PCT, CRP, lymphocyte count, blood glucose, aspartate aminotransferase (AST), and direct bilirubin (all p-values < 0.05). Regarding blood count-derived inflammatory markers, the levels of NLR, LCR, CAR, SIRI, and SII differed significantly between the two groups (all p-values < 0.05).

### The predictive value of presepsin for the severity and prognosis of COVID-19 patients

**Fig. 2:**
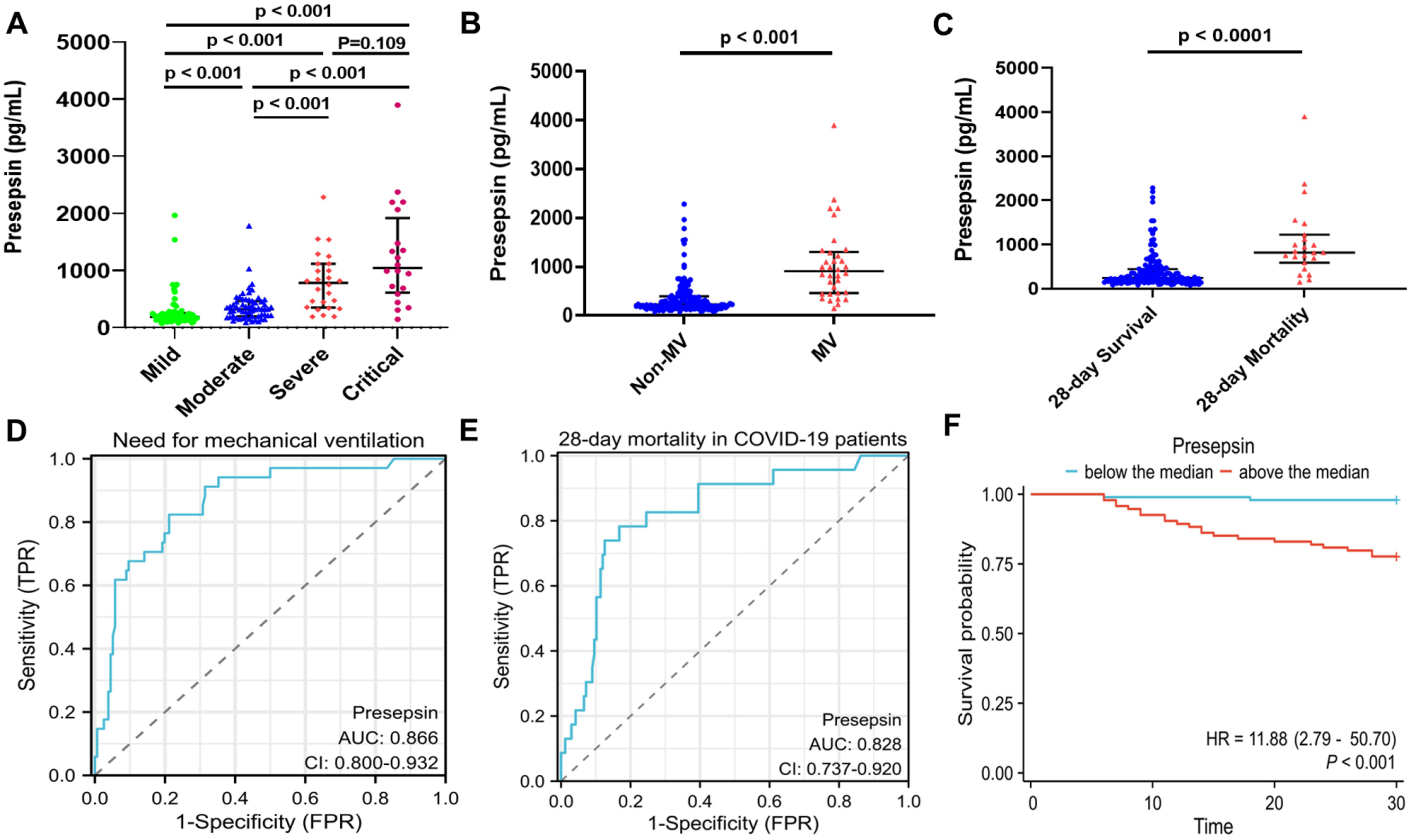
The predictive value of Presepsin for the severity of illness and 28-day mortality in COVID-19 patients. (**A**) Comparison of Presepsin levels among different severity groups of COVID-19 patients. (**B**) Comparison of Presepsin levels between the non-mechanical ventilation and mechanical ventilation groups. (**C**) Comparison of Presepsin levels between the 28-day survival and death groups. Data are displayed as a median with interquartile range (IQR) and were compared using the Mann-Whitney U test. Multiple samples were compared using the non-parametric Kruskal-Wallis test. (**D**) Receiver Operating Characteristic (ROC) curve of Presepsin for predicting the need for mechanical ventilation in COVID-19 patients, with an area under the ROC curve (AUC) of 0.866 (95% confidence interval [CI]: 0.800-0.932). (**E**) ROC curve of Presepsin for predicting 28-day mortality in COVID-19 patients, with an AUC of 0.828 (95% confidence interval [CI]: 0.737–0.920). (**F**) Kaplan-Meier curve for patients divided into two groups based on the median Presepsin level: above-median group and below-median group, for 28-day survival. A p-value < 0.05 was considered significant

The study found that Presepsin levels were associated with the severity of illness in COVID-19 patients, with higher levels observed in patients with more severe conditions (Fig. 2A). Patients requiring mechanical ventilation had significantly higher Presepsin levels compared to those not requiring mechanical ventilation (Fig. 2B). Additionally, patients who died within 28 days had higher Presepsin levels (Fig. 2C). Presepsin demonstrated good predictive value for the need for mechanical ventilation, with an area under the ROC curve of 0.866 (95% confidence interval [CI]: 0.800-0.932) (Fig. 2D), and for 28-day mortality in patients, with an area under the ROC curve of 0.828 (95% confidence interval [CI]: 0.737–0.920) (Fig. 2E). When patients were grouped based on the median value, those above the median value had a higher risk of mortality within 28 days compared to those below the median value (p-value < 0.05) (Fig. 2F).

### The correlations between presepsin and age, clinical scoring systems, and laboratory inflammatory markers

**Fig. 3:**
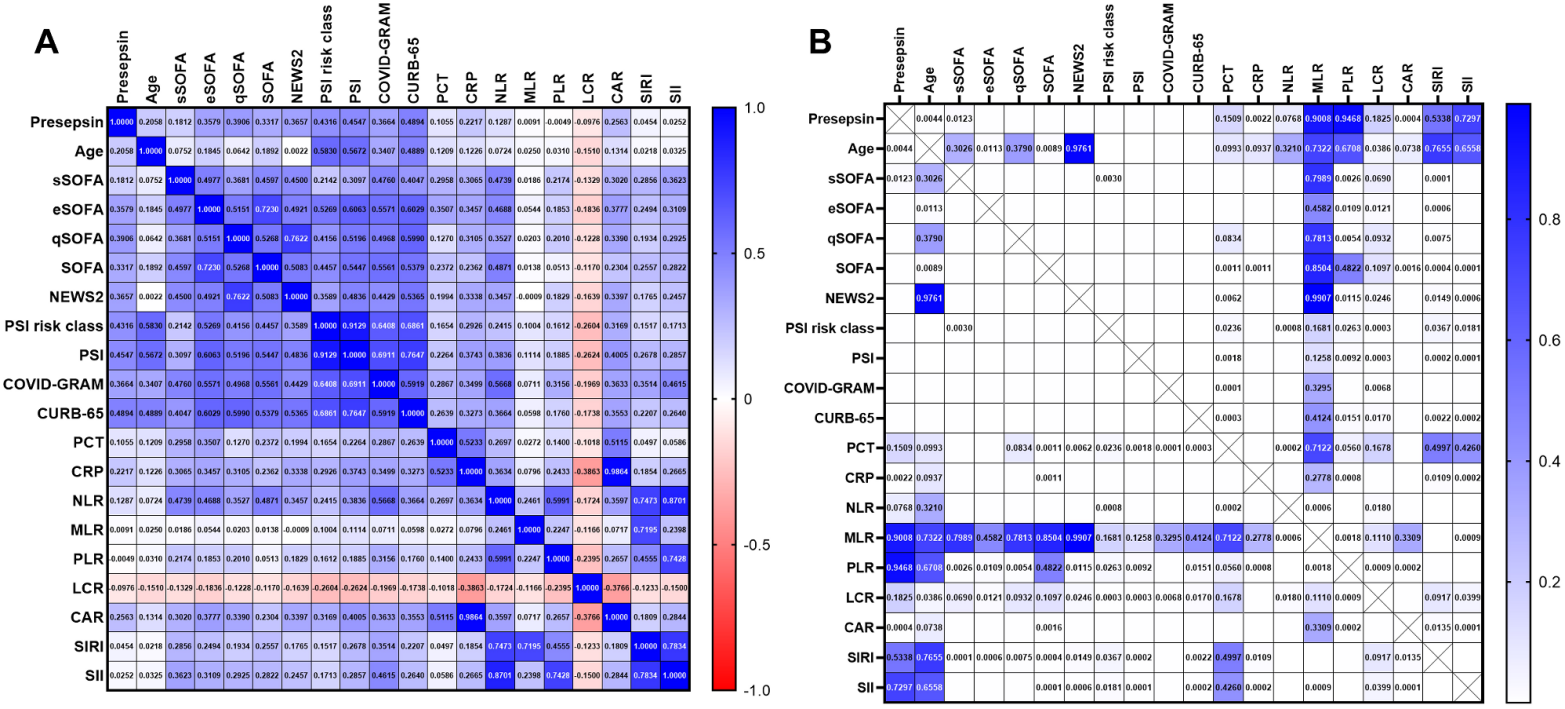
Heatmap showing the correlation between Presepsin and age, clinical scores, and inflammation markers. (**A**) The values are presented as Spearman‘s correlation coefficient (r) for a sample of 190 runners regarding Presepsin. The colormap ranges from 1 to -1, with blue indicating the highest value and red indicating the lowest value. (**B**) The Heatmap of corresponding p-values.The colormap ranges from 0 to 1, with blue representing the largest value and white representing the smallest value. White cells without numerical values indicate that the p-value is smaller than 0.0001, indicating a highly significant correlation. *Abbreviations* s, e, q SOFA, simplified, early, quick sequential organ failure assessment; NEWS2, National Early Warning Score 2; PSI, Pneumonia Severity Index; PCT, Procalcitonin; CRP, C-reactive protein; NLR, Neutrophil-to-lymphocyte ratio; MLR, Monocyte-to-lymphocyte ratio; PLR, Platelet-to-lymphocyte ratio; LCR, Lymphocyte-to-C-reactive protein ratio; CAR, C-reactive protein-to-albumin ratio; SIRI, Systemic inflammation response index; SII: Systemic inflammation index. SIRI = (Neutrophil count × Monocyte count) / Lymphocyte count; SII = (Neutrophil count × Platelet count) / Lymphocyte count

The correlations and corresponding p-values among Presepsin, age, clinical scoring systems, and laboratory inflammatory markers, totaling 20 parameters, are displayed in a heatmap as shown in Fig. 3. Among these parameters, Presepsin exhibited a significant correlation with Age (*r* = 0.206, *p* = 0.0044). Additionally, significant correlations were observed between Presepsin and the following clinical scoring systems and laboratory inflammatory markers: sSOFA (*r* = 0.181, *p* = 0.0123), eSOFA (*r* = 0.358, *p* < 0.0001), qSOFA (*r* = 0.391, *p* < 0.0001), SOFA (*r* = 0.332, *p* < 0.0001), NEWS2 (*r* = 0.366, *p* < 0.0001), PSI risk class (*r* = 0.432, *p* < 0.0001), PSI (*r* = 0.455, *p* < 0.0001), COVID-GRAM (*r* = 0.366, *p* < 0.0001), CURB-65 (*r* = 0.489, *p* < 0.0001), CRP (*r* = 0.222, *p* = 0.0022), and CAR (*r* = 0.256, *p* = 0.0004), as shown in Table 2.

**Table 2:** Correlation between Presepsin, age, clinical score systems, and laboratory markers of inflammation

Variables	Presepsin	
	r-value	p-value
Age	0.206	0.0044*
sSOFA	0.181	0.0123*
eSOFA	0.358	< 0.0001*
qSOFA	0.391	< 0.0001*
SOFA	0.332	< 0.0001*
NEWS2	0.366	< 0.0001*
PSI risk class	0.432	< 0.0001*
PSI	0.455	< 0.0001*
COVID-GRAM	0.366	< 0.0001*
CURB-65	0.489	< 0.0001*
PCT	0.105	0.1509
CRP	0.222	0.0022*
NLR	0.129	0.0768
MLR	0.009	0.9008
PLR	-0.005	0.9468
LCR	-0.0976	0.1825
CAR	0.256	0.0004*
SIRI	0.0454	0.5338
SII	0.0252	0.7297

The values are presented as Spearman’s r of 190 runners for Presepsin. *Abbreviations* s, e, q SOFA, simplified, early, quick sequential organ failure assessment; NEWS2, National Early Warning Score 2; PSI, Pneumonia Severity Index; PCT, Procalcitonin; CRP, C-reactive protein; NLR, Neutrophil-to-lymphocyte ratio; MLR, Monocyte-to-lymphocyte ratio; PLR, Platelet-to-lymphocyte ratio; LCR, Lymphocyte-to-C-reactive protein ratio; CAR, C-reactive protein-to-albumin ratio; SIRI, Systemic inflammation response index; SII: Systemic inflammation index. SIRI = (Neutrophil count × Monocyte count) / Lymphocyte count; SII = (Neutrophil count × Platelet count) / Lymphocyte count

*p-value < 0.05 was considered significant

### The predictive value of clinical scoring systems and laboratory inflammatory markers for 28-day mortality in COVID-19 patients

**Fig. 4:**
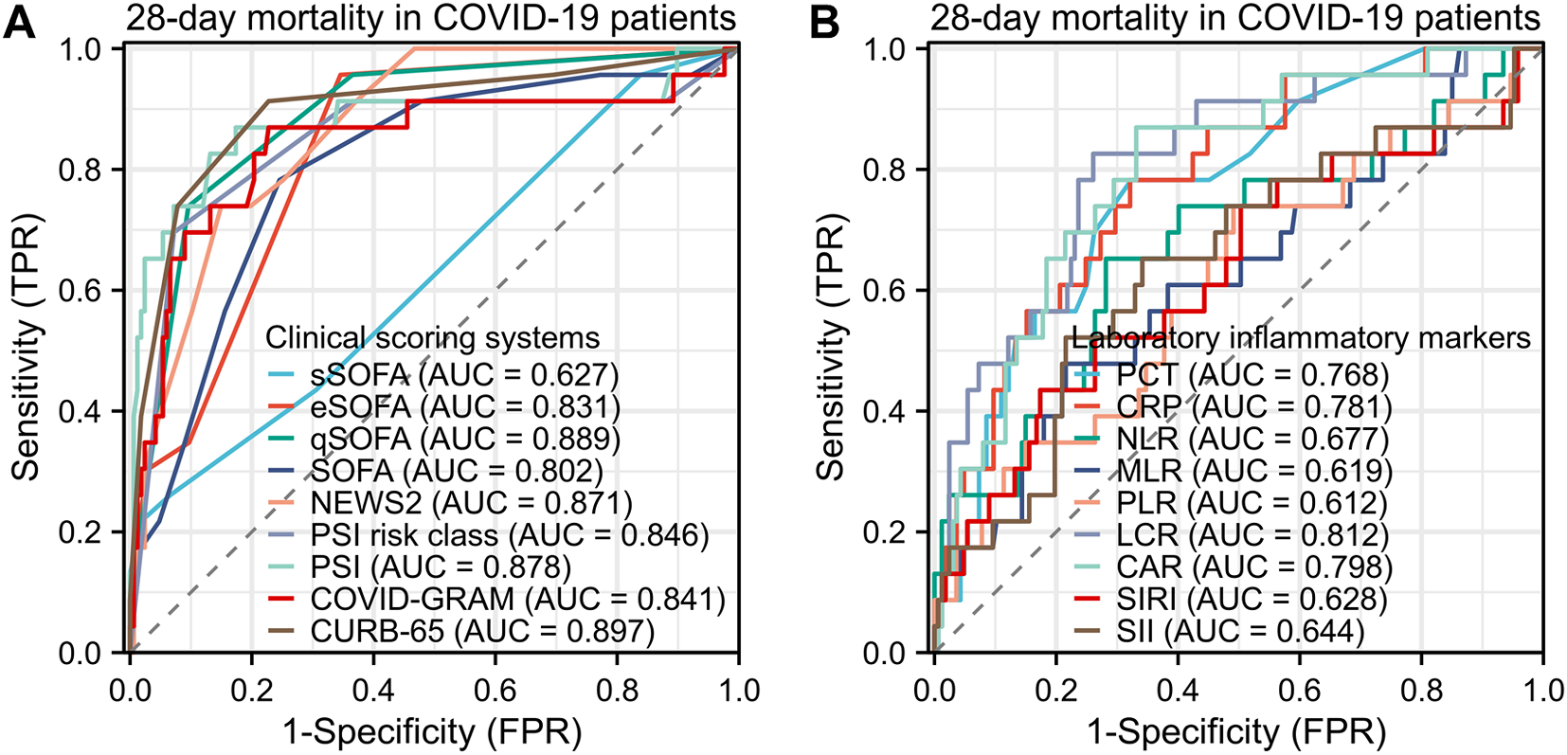
Predictive ability of clinical scores and inflammatory markers for 28-day mortality in COVID-19 patients. (**A**) Receiver Operating Characteristic (ROC) curves for different clinical prediction scores in predicting 28-day mortality in COVID-19 patients. The area under the curve (AUC) for sSOFA was 0.627 (95% confidence interval [CI]: 0.508–0.746), eSOFA, AUC was 0.831 (95% CI: 0.761–0.900); qSOFA, AUC was 0.889 (95% CI: 0.823–0.955); SOFA, AUC was 0.802 (95% CI: 0.705–0.900); NEWS2, AUC was 0.871 (95% CI: 0.808–0.934); PSI risk class, AUC was 0.846 (95% CI: 0.737–0.954); PSI, AUC was 0.878 (95% CI: 0.773–0.984); COVID-GRAM, AUC was 0.841 (95% CI: 0.730–0.953); CURB-65, AUC was 0.897 (95% CI: 0.817–0.978). (**B**) ROC curves for different laboratory inflammatory markers in predicting 28-day mortality in COVID-19 patients. PCT, AUC was 0.768 (95% CI: 0.670–0.866); CRP, AUC was 0.781 (95% CI: 0.685–0.878); NLR, AUC was 0.677 (95% CI: 0.546–0.808); MLR, AUC was 0.619 (95% CI: 0.488–0.750); PLR, AUC was 0.612 (95% CI: 0.480–0.743); LCR, AUC was 0.812 (95% CI: 0.716–0.907); CAR, AUC was 0.798 (95% CI: 0.706–0.890); SIRI, AUC was 0.628 (95% CI: 0.492–0.764); SII, AUC was 0.644 (95% CI: 0.513–0.775). *Abbreviations* TPR: true positive rate; FPR: false positive rate; s, e, q SOFA, simplified, early, quick sequential organ failure assessment; NEWS2, National Early Warning Score 2; PSI, Pneumonia Severity Index; PCT, Procalcitonin; CRP, C-reactive protein; NLR, Neutrophil-to- lymphocyte ratio; MLR, Monocyte-to-lymphocyte ratio; PLR, Platelet-to-lymphocyte ratio; LCR, Lymphocyte-to-C-reactive protein ratio; CAR, C-reactive protein-to-albumin ratio; SIRI, Systemic inflammation response index; SII: Systemic inflammation index. SIRI = (Neutrophil count × Monocyte count) / Lymphocyte count; SII = (Neutrophil count × Platelet count) / Lymphocyte count

Clinical scoring systems and laboratory inflammatory markers also have predictive value for 28-day mortality in COVID-19 patients. Among the clinical scoring systems, the CURB-65 demonstrated the best predictive performance, with an AUC of 0.897 (95% CI: 0.817–0.978) Among the inflammatory markers, LCR performed the best, with an AUC of 0.812 (95% CI: 0.716–0.907), as shown in Fig. 4.

We performed screening using the LASSO binary logistic regression model, and ultimately, three clinical scoring models (CURB-65, PSI, qSOFA) and four inflammation-related markers (PCT, CAR, LCR, NLR) were selected (Figure s1). Additionally, we were also interested in NEWS2, COVID-GRAM and CRP. Therefore, we ultimately selected a total of 10 parameters. The predictive value of these parameters is presented in Table 3.

**Table 3:** Predicted value information of different variable parameters for 28-day mortality in COVID-19 patients

Variables	Cut off value	Sensitivity (95% CI)	Specificity (95% CI)	PPV (95% CI)	NPV (95% CI)	Accuracy (95% CI)	Youden index
Presepsin, pg/mL	562	0.78 (0.56–0.93)	0.83 (0.77–0.89)	0.39 (0.25–0.55)	0.97 (0.92–0.99)	0.83 (0.76–0.88)	0.61
qSOFA	1.5	0.74 (0.52–0.90)	0.90 (0.85–0.94)	0.52 (0.34–0.69)	0.96 (0.6\92-0.99)	0.88 (0.83–0.92)	0.64
NEWS2	7.5	0.74 (0.52–0.90)	0.85 (0.79–0.90)	0.40 (0.26–0.57)	0.96 (0.91–0.98)	0.84 (0.77–0.88)	0.59
PSI	111	0.87 (0.66–0.97)	0.83 (0.76–0.88)	0.41 (0.30–0.56)	0.98 (0.94-1.00)	0.83 (0.77–0.88)	0.70
COVID-GRAM	146	0.87 (0.66–0.97)	0.77 (0.70–0.83)	0.34 (0.22–0.48)	0.98 (0.94-1.00)	0.78 (0.72–0.84)	0.64
CURB-65	1.5	0.91 (0.72–0.99)	0.77 (0.70–0.83)	0.36 (0.24–0.49)	0.98 (0.95-1.00)	0.79 (0.72–0.84)	0.69
PCT, ng/mL	0.105	0.78 (0.56–0.93)	0.68 (0.60–0.75)	0.25 (0.16–0.37)	0.96 (0.90–0.99)	0.69 (0.62–0.75)	0.46
CRP, mg/L	32.395	0.78 (0.56–0.93)	0.68 (0.60–0.75)	0.25 (0.16–0.37)	0.96 (0.90–0.99)	0.69 (0.62–0.76)	0.46
NLR	5.306	0.65 (0.43–0.84)	0.72 (0.64–0.79)	0.24 (0.14–0.37)	0.94 (0.88–0.97)	0.71 (0.64–0.77)	0.37
CAR	0.859	0.87 (0.66–0.97)	0.67 (0.59–0.74)	0.27 (0.17–0.39)	0.97 (0.92–0.99)	0.69 (0.62–0.76)	0.54
LCR	0.0220	0.83 (0.61–0.95)	0.74 (0.67–0.80)	0.31 (0.20–0.44)	0.97 (0.92–0.99)	0.75 (0.68–0.81)	0.57

*Abbreviations* CI, Confidence Interval; PPV, Positive predictive value; NPV, Negative predictive value; qSOFA, quick sequential organ failure assessment; NEWS2, National Early Warning Score 2; PSI, Pneumonia Severity Index; PCT, Procalcitonin; CRP, C-reactive protein; NLR, Neutrophil-to-lymphocyte ratio; CAR, C-reactive protein-to-albumin ratio; LCR, Lymphocyte-to-C-reactive protein ratio. Youden index = Sensitivity + Specificity – 1

**Fig. 5:**
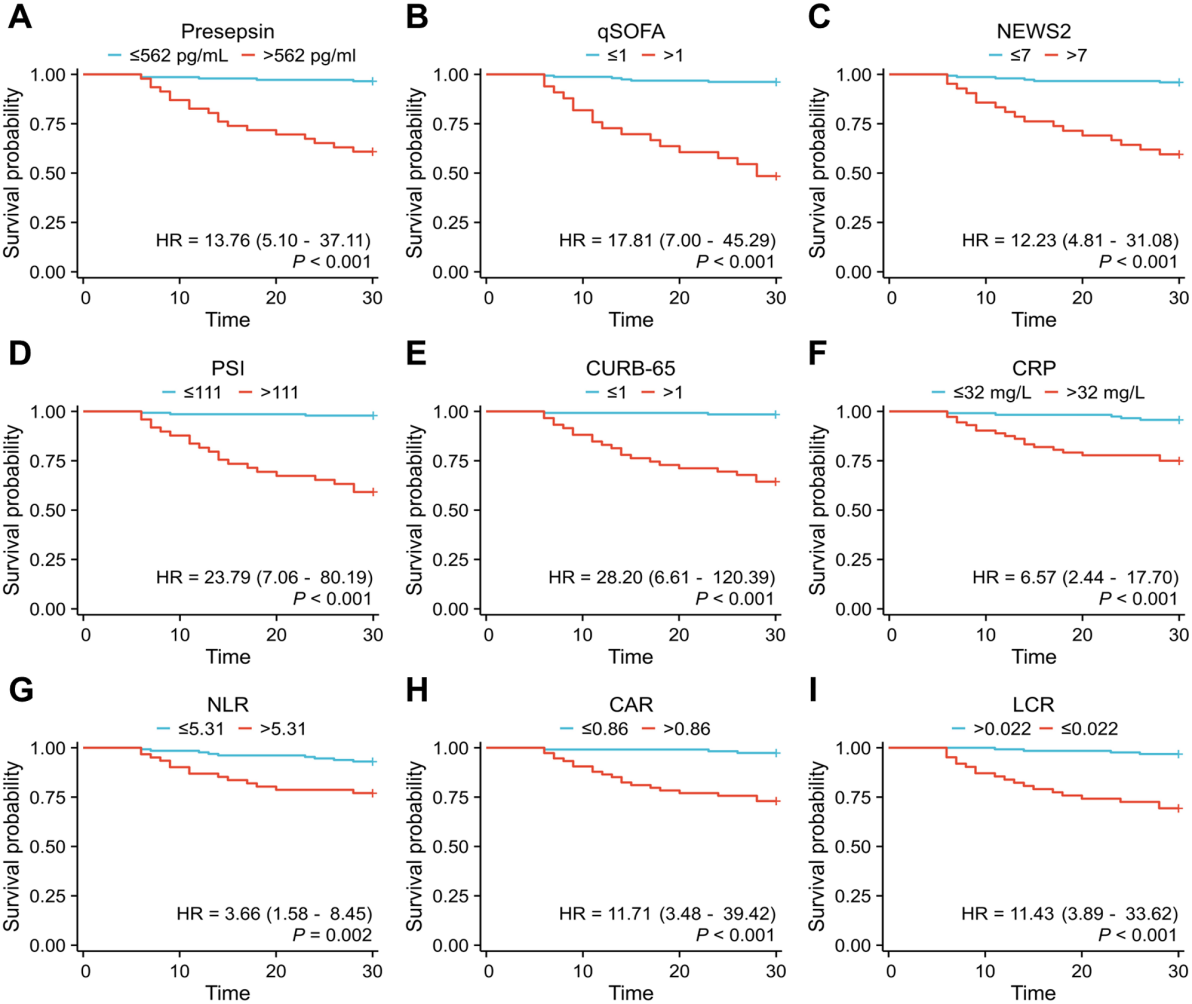
Kaplan-Meier curves for 28-day survival categorized by different parameters. Presepsin (**A**), qSOFA (**B**), NEWS2 (**C**), PSI (**D**), CURB-65 (**E**), CRP (**F**), NLR (**G**), CAR (**H**), and LCR (**I**). *Abbreviations* qSOFA, quick sequential organ failure assessment; NEWS2, National Early Warning Score 2; PSI, Pneumonia Severity Index; CRP, C-reactive protein; NLR, Neutrophil-to-lymphocyte ratio; CAR, C-reactive protein-to-albumin ratio; LCR, Lymphocyte-to-C-reactive protein ratio

Finally, to account for potential confounding factors such as age, diabetes mellitus, malignant tumor, body temperature, respiratory rate, heart rate, PaO2/FiO2, AST, and DBIL, we included these variables as covariates in a multivariable Cox regression analysis. Ultimately, we found that Presepsin, qSOFA, NEWS2, PSI, CURB-65, CRP, NLR, CAR and LCR were the nine independent predictors of 28-day mortality in COVID-19 patients, as shown in Table 4; Fig. 5.

**Table 4:** Risk factors for 28-day Mortality in COVID-19 patients

Variables	No. of 28-day deaths /total (%)	UV			MV	
		HR (95% CI)	P-Value		Adjusted HR (95% CI)	P-Value
**Presepsin, pg/mL**						
≤562	5/144 (3.5)					
>562	18/46 (39.1)	13.7 (5.1,36.9)	< 0.001*		8.5 (2.3,31.3)	0.001*
**qSOFA**						
≤1	6/157 (3.8)					
>1	17/33 (51.5)	17.6 (6.9,44.9)	< 0.001*		9.2 (3.0,28.2)	< 0.001*
**NEWS2**						
≤7	6/148 (4.1)					
>7	17/42 (40.5)	12.2 (4.8,30.9)	< 0.001*		3.6 (1.1,12.6)	0.041*
**PSI**						
≤111	3/141 (2.1)					
>111	20/49 (40.8)	23.6 (7.0,79.7)	< 0.001*		9.2 (2.2,38.8)	0.003*
**COVID-GRAM**						
≤146	4/134 (3.0)					
>146	19/56 (33.9)	13.3 (4.5,39.2)	< 0.001*		3.4 (0.90,13.0)	0.072
**CURB-65**						
≤1	2/131 (1.5)					
>1	21/59 (35.6)	28.0 (6.6,119.7)	< 0.001*		9.1 (1.8,46.8)	0.008*
**PCT, ng/mL**						
≤0.105	5/116 (4.3)					
>0.105	18/71 (25.4)	6.7 (2.5,18.0)	< 0.001*		2.9 (0.77,10.59)	0.115
**CRP, mg/L**						
≤32	5/116 (4.3)					
>32	18/72 (25.0)	6.5 (2.4,17.6)	< 0.001*		6.1 (1.7,21.5)	0.005*
**NLR**						
≤5.31	9/129 (7.0)					
>5.31	14/61 (23.0)	3.6 (1.6,8.4)	0.002		4.33 (1.47,12.78)	0.008*
**CAR**						
≤0.86	3/113 (2.7)					
>0.86	20/74 (27.0)	11.7 (3.5,39.3)	< 0.001*		10.0 (2.19,45.83)	0.003*
**LCR**						
≤0.022	19/62 (30.6)					
>0.022	4/126 (3.2)	0.088 (0.03,0.26)	< 0.001*		0.163 (0.049,0.546)	0.003*

Performed with age, diabetes mellitus, malignant tumor, body temperature, respiratory rate, heart rate, PaO_2_/FiO_2,_ AST, DBIL as covariates. Cox regression analyses was performed on 190 COVID-19 patients. *Abbreviations* PaO_2_, oxygen tension; FiO_2_, fraction of inspired oxygen; AST, Aspartate aminotransferase; DBIL, Direct bilirubin; UV, Univariate Analysis; MV, Multivariate Analysis; HR, Hazard Ratio; CI, Confidence Interval; qSOFA, quick sequential organ failure assessment; NEWS2, National Early Warning Score 2; PSI, Pneumonia Severity Index; PCT, Procalcitonin; CRP, C-reactive protein; NLR, Neutrophil-to-lymphocyte ratio; CAR, C-reactive protein-to-albumin ratio; LCR, Lymphocyte-to-C-reactive protein ratio

*p-value < 0.05 was considered significant

### The combined predictive value of presepsin with clinical scoring systems and laboratory inflammatory markers for 28-day mortality in COVID-19 patients

**Fig. 6:**
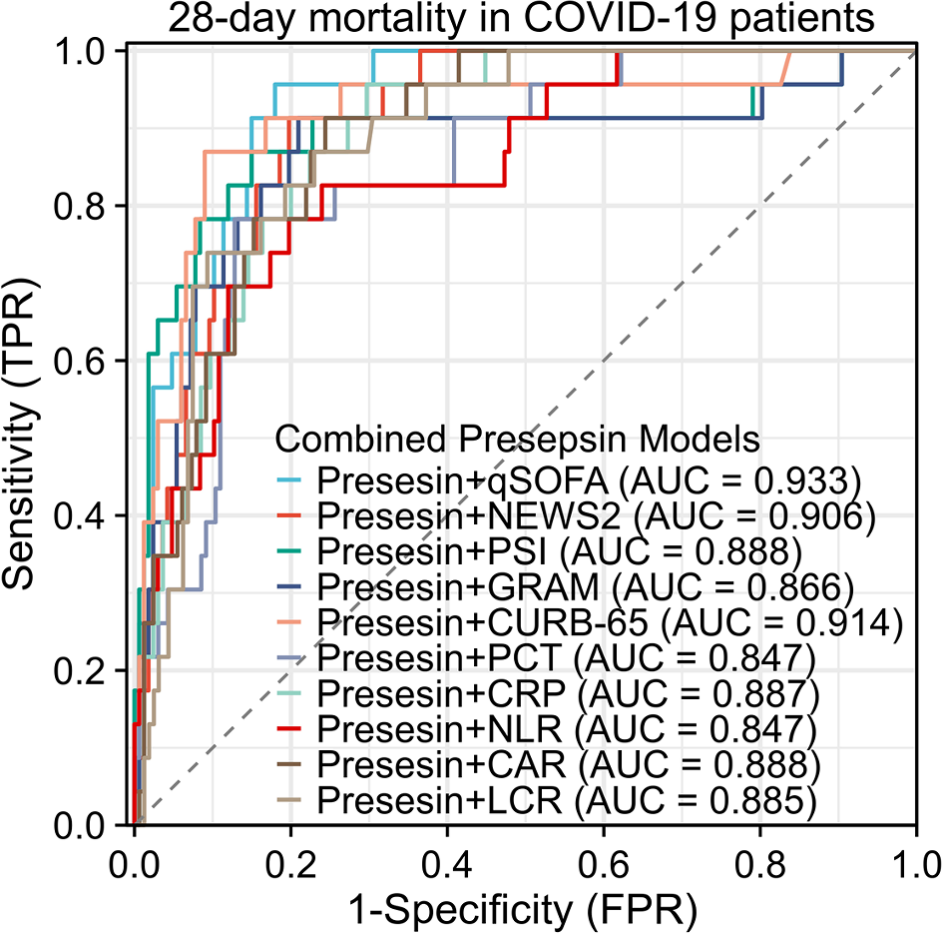
Presepsin’s predictive ability for 28-day mortality in COVID-19 patients with clinical scores or inflammatory markers. The area under the curve (AUC) for Presesin + qSOFA was 0.933 (95% confidence interval [CI]: 0.893–0.972), Presesin + NEWS2, AUC was 0.906 (95% CI: 0.856–0.955); Presesin + PSI, AUC was 0.888 (95% CI: 0.789–0.987); Presesin + GRAM, AUC was 0.866 (95% CI: 0.767–0.966); Presesin + CURB-65, AUC was 0.914 (95% CI: 0.840–0.988); Presesin + PCT, AUC was 0.847 (95% CI: 0.770–0.925); Presesin + CRP, AUC was 0.887 (95% CI: 0.831–0.943); Presesin + NLR, AUC was 0.847 (95% CI: 0.765–0.929); Presesin + CAR, AUC was 0.888 (95% CI: 0.833–0.944); Presesin + LCR, AUC was 0.885 (95% CI: 0.825–0.945). *Abbreviations* qSOFA, quick sequential organ failure assessment; NEWS2, National Early Warning Score 2; PSI, Pneumonia Severity Index; PCT, Procalcitonin; CRP, C-reactive protein; NLR, Neutrophil-to-lymphocyte ratio; CAR, C-reactive protein-to-albumin ratio; LCR, Lymphocyte-to-C-reactive protein ratio

We combined Presepsin with the selected 10 parameters to compare the predictive efficacy for the 28-day prognosis of COVID-19 patients. It was found that Presepsin + qSOFA had the best predictive performance, with an area under the curve (AUC) of 0.933 (95% confidence interval [CI]: 0.893–0.972). Presepsin + CURB-65 ranked second with an AUC of 0.914 (95% CI: 0.840–0.988), followed by Presepsin + NEWS2 with an AUC of 0.906 (95% CI: 0.856–0.955) as the third best predictor. Among the inflammation-related markers, Presepsin + CAR exhibited the best predictive performance with an AUC of 0.888 (95% CI: 0.833–0.944). As shown in Table 5; Fig. 6.

**Table 5:** AUC for predicting COVID-19 mortality using various parameters and models

Variables	AUC	Standard error	P value	95% confidence interval	
				Lower limit	Upper limit
Presepsin	0.829	0.046	*p* < 0.001*	0.737	0.920
qSOFA	0.889	0.036	*p* < 0.001*	0.823	0.955
NEWS2	0.871	0.033	*p* < 0.001*	0.808	0.934
PSI	0.878	0.054	*p* < 0.001*	0.773	0.984
COVID-GRAM	0.841	0.056	*p* < 0.001*	0.730	0.953
CURB-65	0.897	0.042	*p* < 0.001*	0.817	0.978
PCT	0.768	0.049	*p* < 0.001*	0.670	0.866
CRP	0.781	0.048	*p* < 0.001*	0.686	0.876
NLR	0.677	0.066	*p* < 0.001*	0.546	0.808
CAR	0.798	0.046	*p* < 0.001*	0.706	0.890
LCR	0.812	0.048	*p* < 0.001*	0.716	0.907
Presepsin + qSOFA	0.933	0.020	*p* < 0.001*	0.893	0.972
Presepsin + NEWS2	0.906	0.025	*p* < 0.001*	0.856	0.955
Presepsin + PSI	0.888	0.051	*p* < 0.001*	0.789	0.987
Presepsin + COVID-GRAM	0.866	0.050	*p* < 0.001*	0.767	0.966
Presepsin + CURB-65	0.914	0.037	*p* < 0.001*	0.840	0.988
Presepsin + PCT	0.847	0.039	*p* < 0.001*	0.770	0.925
Presepsin + CRP	0.887	0.028	*p* < 0.001*	0.831	0.942
Presepsin + NLR	0.847	0.042	*p* < 0.001*	0.765	0.929
Presepsin + CAR	0.888	0.029	*p* < 0.001*	0.833	0.944
Presepsin + LCR	0.885	0.030	*p* < 0.001*	0.825	0.945

*Abbreviations* AUC, area under the receiver operating characteristic curve; qSOFA, quick sequential organ failure assessment; NEWS2, National Early Warning Score 2; PSI, Pneumonia Severity Index; PCT, Procalcitonin; CRP, C-reactive protein; NLR, Neutrophil-to-lymphocyte ratio; CAR, C-reactive protein-to-albumin ratio; LCR, Lymphocyte-to-C-reactive protein ratio

**Fig. 7:**
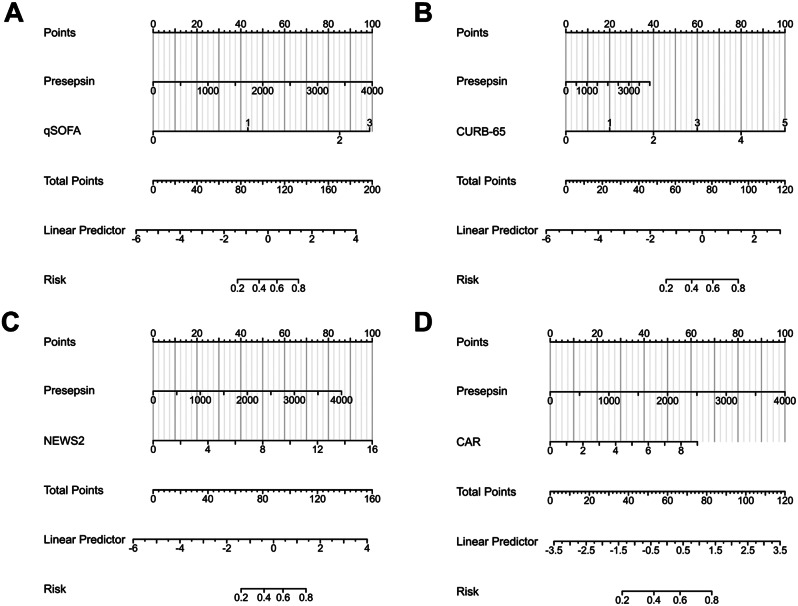
Nomograms for predicting COVID-19 mortality using combined Presepsin models. **A**: Presepsin + qSOFA; **B**: Presepsin + CURB-65; **C**: Presepsin + NEWS2; **D**: Presepsin + CAR. Nomograms are useful for individual prediction and risk stratification, providing an intuitive way to estimate probabilities or predict outcomes based on multiple variables. For each predictor variable, locate the corresponding value on its scale and record the point indicated by that position. Sum up the points obtained for each predictor variable. Locate the total points on the prediction scale of the nomogram to estimate the predicted outcome or probability associated with the model. For example, in Fig. 7A, when a patient has a Presepsin level of 1000, corresponding to 25 points, and a qSOFA score of 2, corresponding to 87 points, the total score is 112, indicating a 60% probability of death within 28 days for that patient. *Abbreviations* qSOFA, quick sequential organ failure assessment; NEWS2, National Early Warning Score 2; CAR, C-reactive protein-to-albumin ratio

Finally, for the convenience of clinical decision-making, we constructed a nomogram for the combined model of Presepsin and these four indicators (Fig. 7). The calibration curve of the nomogram for predicting the risk of 28-day mortality in COVID-19 patients is presented in Figure s2, showing good agreement between the predicted probabilities of 28-day mortality by the nomogram and the observed probabilities (all p-values > 0.05). The decision curve analysis (DCA) of the nomogram model is displayed in Figure s3, covering a threshold probability range from 1 to 90%. These results indicate that the nomogram’s calibration was acceptable and the model was reliable for clinical utility.

## Discussion

Early identification and intervention in critically ill patients are crucial for reducing mortality in COVID-19 patients. Our study found a correlation between Presepsin levels in the plasma of COVID-19 patients and the severity of the disease. Additionally, Presepsin showed good predictive value for the prognosis of COVID-19 patients. We also evaluated the predictive performance of various clinical scoring systems and inflammation-related markers for 28-day mortality in COVID-19 patients. We found that NEWS2, PSI, CURB-65, CRP, NLR, CAR, and LCR were independent predictors of 28-day mortality in COVID-19 patients. Combining Presepsin with clinical scoring systems and inflammation-related markers improved the prediction of adverse outcomes in COVID-19 patients. Finally, we selected four combinations with the best predictive performance and developed four nomograms to assist clinical decision-making.

Presepsin is considered a promising novel biomarker for sepsis and holds significant value in risk stratification and prognosis assessment for septic patients [22, 24, 34, 35]. Mild COVID-19 patients exhibit a milder inflammatory response, while severe COVID-19 patients experience a more pronounced inflammatory response, including the occurrence of an immune cytokine storm [36]. The innate immune response plays a significant role in the immune cytokine storm observed in severe COVID-19 patients [37, 38]. Plasma levels of presepsin can be considered as an indicator of activating innate immune cells in response to invading pathogens [22]. Presepsin can reflect the severity of patients’ inflammatory response. Therefore, the Presepsin levels are significantly elevated in severe COVID-19 patients compared to mild cases. C-reactive protein (CRP) is a commonly used clinical marker of inflammation. In the correlation analysis (Table 2; Fig. 3), we observed a significant correlation between Presepsin and CRP (*r* = 0.222, *p* = 0.0022). This further supports our research conclusion. Recent meta-analysis studie have indicated that Presepsin is a promising biomarker that can accurately reflect the severity of COVID-19 [25]. Since the outbreak of the novel coronavirus in 2020, studies have found that Presepsin levels are higher in hospitalized patients who died compared to those who survived [21, 39, 40]. Presepsin levels have also been found to correlate with the need for ICU admission, ICU length of stay, and total hospital stay. Additionally, research has shown that Presepsin levels increase in patients requiring invasive mechanical ventilation and decrease as symptoms improve in severely ill patients [41, 42]. However, these studies had small sample sizes or relied on indirect evidence through bioinformatics analysis. In 2022, a study conducted in Saudi Arabia and Misr International Hospital, Egypt, included 202 patients. The study found a significant positive correlation between Presepsin (PSP) and PSI scores, as well as inflammation markers such as NLR, D-dimer, ferritin, CRP, and ESR. Additionally, PSP showed superior predictive value for in-hospital mortality in COVID-19 pneumonia compared to NLR, ferritin, and CRP. The study reported a mortality rate of 26/202 (12.9%), which is comparable to our study’s mortality rate of 23/190 (12.1%). The optimal cutoff value in that study was 775 pg/mL, while our study had a lower cutoff value of 562 pg/mL [43]. Our study focused on a population from mainland China and evaluated the value of Presepsin in combination with clinical scoring systems and inflammation-related markers for the prognosis of COVID-19 patients. We also developed nomograms to assist clinical decision-making.

Clinical prediction scores hold significant value in clinical practice, and PSI and CURB-65 are widely used assessment systems for the severity of pneumonia. Since the outbreak of COVID-19, they have been employed to predict the prognosis of COVID-19 patients. Current research demonstrates that PSI and CURB-65 have good predictive value for COVID-19 mortality, with AUCs ranging from 0.79 to 0.91 and 0.79 to 0.88, respectively [17, 44–46]. In our study, the AUC for PSI was 0.878, and for CURB-65 it was 0.897, which is consistent with previous research findings. The NEWS2 and qSOFA have been proposed as candidate scores for predicting the prognosis of COVID-19 in situations with limited medical resources [46, 47]. One study has indicated that the AUC of NEWS2 can reach 0.87 [19], which is similar to our research findings. In our study, the AUC for NEWS2 was 0.871 (95% CI: 0.808–0.934). Some studies have indicated that qSOFA has good predictive value for the prognosis of COVID-19 patients [46, 48, 49]. In our study, qSOFA had an AUC of 0.889 (95% CI: 0.823–0.955). Additionally, the combination of qSOFA with Presepsin showed the highest value. This may be attributed to its high specificity. COVID-GRAM was initially proposed by Chinese scholars and has shown good predictive value for COVID-19 [33]. The AUC in both the development and validation groups can reach 0.88. In our study, the AUC for COVID-GRAM was 0.841 (95% CI: 0.730–0.953).

Immune response and cytokine storm play important roles in the pathophysiological mechanisms of COVID-19 patients, and immune markers are used to predict patient prognosis [50–53]. C-reactive protein (CRP) and procalcitonin (PCT) are classical indicators of inflammatory response, and neutrophil-to-lymphocyte ratio (NLR) is also highly regarded [54–59]. In our study, CRP (AUC was 0.781) and PCT (AUC was 0.768) showed better predictive efficacy for 28-day mortality in COVID-19 patients compared to NLR (AUC was 0.677). Our study also evaluated the predictive value of novel serum biomarkers for the prognosis of COVID-19 patients. In our study, we found that LCR (AUC was 0.812) and CAR (AUC was 0.798) demonstrated good predictive value. However, MLR, PLR, SII, and SIRI showed lower predictive value (AUCs were all less than 0.7). See Fig. 4 for details. CAR is considered a more reliable marker for assessing inflammation status and an independent risk factor for cardiovascular and infectious diseases. Recent studies have found that CAR is an independent predictor of mortality in COVID-19 patients [60, 61]. One study found that LCR can distinguish the severity of COVID-19 in patients and can be used as an auxiliary screening tool for hospital admission and ICU admission [62, 63]. Currently, there is limited research available on these indicators, and even fewer studies that compare these indicators in detail [61].

In conclusion, clinical prediction scores, inflammation markers, and their derived novel prognostic indicators have significant clinical utility in predicting the prognosis of COVID-19 patients. Our study found a good correlation between Presepsin and these clinical scoring systems and inflammation markers (Fig. 3; Table 2). Furthermore, combining Presepsin with clinical prediction scores or inflammation markers can further enhance the predictive value. Combining clinical prediction scores with new laboratory indicators may be a future research direction [64, 65].

Our study has several limitations. Firstly, it was a single-center retrospective study, and the nature of such studies may lead to some unavoidable biases. Secondly, our sample size was still small, mainly due to the difficulty in obtaining blood samples from enrolled patients. Thirdly, although we developed a clinical prediction model in our study, we did not perform external validation. Fourthly, the primary outcome of our study was the 28-day mortality rate. However, the condition of some patients may have changed. Long-term survival outcomes require further research. Fifthly, we did not conduct dynamic monitoring and tracking of Presepsin levels in patients. Incorporating dynamic monitoring of Presepsin levels with changes in clinical conditions may have more meaningful implications for predicting disease progression [66]. However, this is an aspect we plan to address in our future research. We look forward to larger-scale, multicenter studies in the future to validate our findings.

## Conclusion

This study found a correlation between Presepsin levels and the severity of COVID-19 in patients. Presepsin showed good predictive value for 28-day mortality in patients, and combining it with clinical scoring systems and inflammation markers further enhanced the ability to assess patient prognosis. In the future, we anticipate large-scale multicenter studies to validate our research findings.

### Electronic supplementary material

Below is the link to the electronic supplementary material.

Supplementary Material 1

## Data Availability

The datasets used and/or analyzed during the current study are available from the corresponding author on reasonable request.

## Abbreviations

ARDS: acute respiratory distress syndrome
ESR: erythrocyte sedimentation rate
sCD14: soluble CD14
TLR: Toll-like receptor
LPS: lipopolysaccharide
COPD: Chronic Obstructive Pulmonary Disease
RR: respiratory rate
HR: heart rate
SBP: systolic blood pressure
PaCO2: arterial carbon dioxide tension
PaO2: oxygen tension
SpO2: peripheral oxygen saturation
FiO2: fraction of inspired oxygen
sSOFA: Simplified sequential organ failure assessment
eSOFA: early sequential organ failure assessment
qSOFA: quick sequential organ failure assessment
SOFA: sequential organ failure assessment
NEWS2: National Early Warning Score 2
PSI: Pneumonia Severity Index
PCT: Procalcitonin
CRP: C-reactive protein
HGB: Hemoglobin
WBC: White blood cell
BCDIMs: blood count-derived inflammatory markers
INR: International normalized ratio
ALT: Alanine aminotransferase
AST: Aspartate aminotransferase
TBIL: Total bilirubin
DBIL: Direct bilirubin
EDTA: ethylenediaminetetraacetic acid
ICU: intensive care unit
NLR: Neutrophil-to-lymphocyte ratio
MLR: Monocyte-to-lymphocyte ratio
PLR: Platelet-to-lymphocyte ratio
LCR: Lymphocyte-to-C-reactive protein ratio
CAR: C-reactive protein-to-albumin ratio
SIRI: Systemic inflammation response index
SII: Systemic inflammation index. SIRI = (Neutrophil count × Monocyte count) / Lymphocyte count
SII: = (Neutrophil count × Platelet count) / Lymphocyte count
SD: standard deviation
IQR: interquartile range
ROC: Receiver Operating Characteristic
LASSO: least absolute shrinkage and selection operator
DCA: decision curve analysis

## References

[1] Huang C (2020). Clinical features of patients infected with 2019 novel coronavirus in Wuhan, China. Lancet.

[2] Sachs JD (2022). The Lancet Commission on lessons for the future from the COVID-19 pandemic. Lancet.

[3] Mertoglu C (2022). COVID-19 is more dangerous for older people and its severity is increasing: a case-control study. Med Gas Res.

[4] Rubin EJ (2022). Audio interview: viral evolution and the future of monoclonal antibodies. N Engl J Med.

[5] Xavier Becerra X, Jha A (2023). Project NextGen — defeating SARS-CoV-2 and Preparing. N Engl J Med.

[6] The L (2023). The COVID-19 pandemic in 2023: far from over. Lancet.

[7] Kurban LAS (2022). Predicting severe disease and critical illness on initial diagnosis of COVID-19: simple triage tools. Front Med (Lausanne).

[8] Song H (2023). Electrolyte imbalances as poor prognostic markers in COVID-19: a systemic review and meta-analysis. J Endocrinol Invest.

[9] Buttia C (2023). Prognostic models in COVID-19 infection that predict severity: a systematic review. Eur J Epidemiol.

[10] Tjendra Y (2020). Predicting Disease Severity and Outcome in COVID-19 patients: a review of multiple biomarkers. Arch Pathol Lab Med.

[11] Xue G et al. Novel serological biomarkers for inflammation in predicting disease severity in patients with COVID-19. Int Immunopharmacol, 2020. 89. 10.1016/j.intimp.2020.107065 PMC7532789 33045571

[12] Mertoglu C (2021). How do routine laboratory tests change in coronavirus disease 2019?. Scand J Clin Lab Invest.

[13] Zeng F (2020). Association of inflammatory markers with the severity of COVID-19: a meta-analysis. Int J Infect Dis.

[14] Tahir Huyut M (2022). What is the impact and efficacy of routine immunological, biochemical and hematological biomarkers as predictors of COVID-19 mortality?. Int Immunopharmacol.

[15] Huyut MT, Ilkbahar F (2021). The effectiveness of blood routine parameters and some biomarkers as a potential diagnostic tool in the diagnosis and prognosis of Covid-19 disease. Int Immunopharmacol.

[16] Matono T (2022). Diagnostic accuracy of quick SOFA score and inflammatory biomarkers for predicting community-onset bacteremia. Sci Rep.

[17] Bradley J (2022). Pneumonia Severity Index and CURB-65 score are good predictors of mortality in hospitalized patients with SARS-CoV-2 Community-Acquired Pneumonia. Chest.

[18] Bradley P et al. Utility of established prognostic scores in COVID-19 hospital admissions: multicentre prospective evaluation of CURB-65, NEWS2 and qSOFA. BMJ Open Respir Res, 2020. 7(1). 10.1136/bmjresp-2020-000729 PMC7722817 33293361

[19] De Socio GV (2021). National early warning score 2 (NEWS2) better predicts critical Coronavirus Disease 2019 (COVID-19) illness than COVID-GRAM, a multi-centre study. Infection.

[20] Schirinzi A et al. New insights in Laboratory Testing for COVID-19 patients: looking for the Role and Predictive Value of Human epididymis secretory protein 4 (HE4) and the Innate immunity of the oral cavity and respiratory tract. Microorganisms, 2020. 8(11). 10.3390/microorganisms8111718 PMC7692217 33147871

[21] Zaninotto M (2020). Presepsin in risk stratification of SARS-CoV-2 patients. Clin Chim Acta.

[22] Memar MY, Baghi HB (2019). Presepsin: a promising biomarker for the detection of bacterial infections. Biomed Pharmacother.

[23] Chenevier-Gobeaux C (2015). Presepsin (sCD14-ST), an innate immune response marker in sepsis. Clin Chim Acta.

[24] Liu B (2013). Diagnostic value and prognostic evaluation of Presepsin for sepsis in an emergency department. Crit Care.

[25] Guarino M (2023). Presepsin levels and COVID-19 severity: a systematic review and meta-analysis. Clin Exp Med.

[26] The General Office of the National Health Commission. T.O.o.t.S.A.o.T.C.M., Diagnosis and Treatment protocol for COVID-19 patients (tentative 9 version). https://www.gov.cn/zhengce/zhengceku/2022-03/15/content_5679257.htm(Accessed on March 20, 2022).

[27] Asai N (2019). Efficacy and accuracy of qSOFA and SOFA scores as prognostic tools for community-acquired and healthcare-associated pneumonia. Int J Infect Dis.

[28] Gershengorn HB (2022). Predictive value of sequential organ failure Assessment score across patients with and without COVID-19 infection. Ann Am Thorac Soc.

[29] Gulec T (2023). Can we recognize severe community-acquired pneumonia without pneumonia severity index? Use of modified qSOFA with procalcitonin. Heliyon.

[30] Lim WS (2003). Defining community acquired pneumonia severity on presentation to hospital: an international derivation and validation study. Thorax.

[31] Sligl WI, Marrie TJ (2013). Severe community-acquired pneumonia. Crit Care Clin.

[32] Berastegui-Cabrera J (2023). Prepandemic viral community-acquired pneumonia: diagnostic sensitivity and specificity of nasopharyngeal swabs and performance of clinical severity scores. J Med Virol.

[33] Liang W (2020). Development and validation of a clinical risk score to predict the occurrence of critical illness in hospitalized patients with COVID-19. JAMA Intern Med.

[34] Wang C (2023). Early predictive value of Presepsin for secondary Sepsis and mortality in Intensive Care Unit patients with severe Acute Pancreatitis. Shock.

[35] Park JE et al. Complementary use of Presepsin with the Sepsis-3 Criteria Improved Identification of High-Risk patients with suspected Sepsis. Biomedicines, 2021. 9(9). 10.3390/biomedicines9091076 PMC8469631 34572261

[36] Gursoy B (2021). Cytokine storm in severe COVID-19 pneumonia. J Med Virol.

[37] Mehta P (2020). COVID-19: consider cytokine storm syndromes and immunosuppression. Lancet.

[38] Fajgenbaum DC, June CH (2020). Cytokine Storm N Engl J Med.

[39] Chang Y (2022). Presepsin predicts severity and secondary bacterial infection in COVID-19 by Bioinformatics Analysis. Comput Math Methods Med.

[40] Kim SW (2022). Usefulness of monocyte distribution width and presepsin for early assessment of disease severity in COVID-19 patients. Med (Baltim).

[41] Fukada A (2021). Presepsin as a predictive biomarker of severity in COVID-19: a case series. J Med Virol.

[42] Dell’Aquila P (2021). A simple prognostic score based on troponin and presepsin for COVID-19 patients admitted to the emergency department: a single-center pilot study. Acta Biomed.

[43] Assal HH (2022). Presepsin as a Novel Biomarker in predicting In-hospital mortality in patients with COVID-19 pneumonia. Int J Infect Dis.

[44] Artero A (2021). Severity scores in COVID-19 pneumonia: a Multicenter, Retrospective, Cohort Study. J Gen Intern Med.

[45] Satici C (2020). Performance of pneumonia severity index and CURB-65 in predicting 30-day mortality in patients with COVID-19. Int J Infect Dis.

[46] Fan G et al. Comparison of severity scores for COVID-19 patients with pneumonia: a retrospective study. Eur Respir J, 2020. 56(3). 10.1183/13993003.02113-2020 PMC7366179 32675205

[47] Rudd KE (2018). Association of the Quick Sequential (Sepsis-Related) organ failure Assessment (qSOFA) score with excess hospital mortality in adults with suspected infection in low- and Middle-Income Countries. JAMA.

[48] Ruangsomboon O (2023). The utility of the Rapid Emergency Medicine Score (REMS) compared with three other early warning scores in predicting in-hospital mortality among COVID-19 patients in the emergency department: a multicenter validation study. BMC Emerg Med.

[49] Brajkovic M et al. The Predictive Value of Risk Factors and Prognostic Scores in Hospitalized COVID-19 Patients Diagnostics (Basel), 2023. 13(16). 10.3390/diagnostics13162653 PMC10453362 37627912

[50] Ye Q, Wang B, Mao J (2020). The pathogenesis and treatment of the;Cytokine Storm’ in COVID-19. J Infect.

[51] Huyut MT, Velichko A. Diagnosis and prognosis of COVID-19 Disease using routine blood values and LogNNet neural network. Sens (Basel), 2022. 22(13). 10.3390/s22134820 PMC9269123 35808317

[52] Velichko A et al. Machine learning sensors for diagnosis of COVID-19 Disease using routine blood values for internet of things application. Sens (Basel), 2022. 22(20). 10.3390/s22207886 PMC9610709 36298235

[53] Huyut MT, Huyut Z (2023). Effect of ferritin, INR, and D-dimer immunological parameters levels as predictors of COVID-19 mortality: a strong prediction with the decision trees. Heliyon.

[54] Chi L (2023). Predictive value of C-reactive protein for disease severity and survival in COVID-19 patients: a systematic review and meta-analysis. Clin Exp Med.

[55] Bouayed MZ (2022). C-Reactive protein (CRP): a poor prognostic biomarker in COVID-19. Front Immunol.

[56] Smilowitz NR (2021). C-reactive protein and clinical outcomes in patients with COVID-19. Eur Heart J.

[57] Acedera ML (2023). Age, comorbidities, c-reactive protein and procalcitonin as predictors of severity in confirmed COVID-19 patients in the Philippines. Heliyon.

[58] Kumar A (2022). Procalcitonin as a predictive marker in COVID-19: a systematic review and meta-analysis. PLoS ONE.

[59] Sayah W (2021). Interleukin-6, procalcitonin and neutrophil-to-lymphocyte ratio: potential immune-inflammatory parameters to identify severe and fatal forms of COVID-19. Cytokine.

[60] Xu J (2023). Nomogram-based prediction model for survival of COVID-19 patients: a clinical study. Heliyon.

[61] Zavalaga-Zegarra HJ et al. C-Reactive protein-to-albumin ratio and clinical outcomes in COVID-19 patients: a systematic review and Meta-analysis. Trop Med Infect Dis, 2022. 7(8). 10.3390/tropicalmed7080186 PMC9414550 36006278

[62] Erdogan A, Can FE, Gonullu H (2021). Evaluation of the prognostic role of NLR, LMR, PLR, and LCR ratio in COVID-19 patients. J Med Virol.

[63] Zhang JN (2022). Lymphocyte-C-reactive protein ratio can differentiate disease severity of COVID-19 patients and serve as an assistant screening tool for hospital and ICU admission. Front Immunol.

[64] Sadhu S (2023). IL-9 aggravates SARS-CoV-2 infection and exacerbates associated airway inflammation. Nat Commun.

[65] Chen YM (2020). Blood molecular markers associated with COVID-19 immunopathology and multi-organ damage. EMBO J.

[66] Behnes M (2014). Diagnostic and prognostic utility of soluble CD 14 subtype (presepsin) for severe sepsis and septic shock during the first week of intensive care treatment. Crit Care.

