# Supplementary material for: Presepsin as a prognostic biomarker in COVID-19 patients: combining clinical scoring systems and laboratory inflammatory markers for outcome prediction
Source: Virol J. 2024 Apr 26;21:96. doi: 10.1186/s12985-024-02367-1 (PMC11046891; doi:10.1186/s12985-024-02367-1)
Supplement: Supplementary file 1 — Supplementary Material 1 [file 12985_2024_2367_MOESM1_ESM.docx]

**Additional file 1**


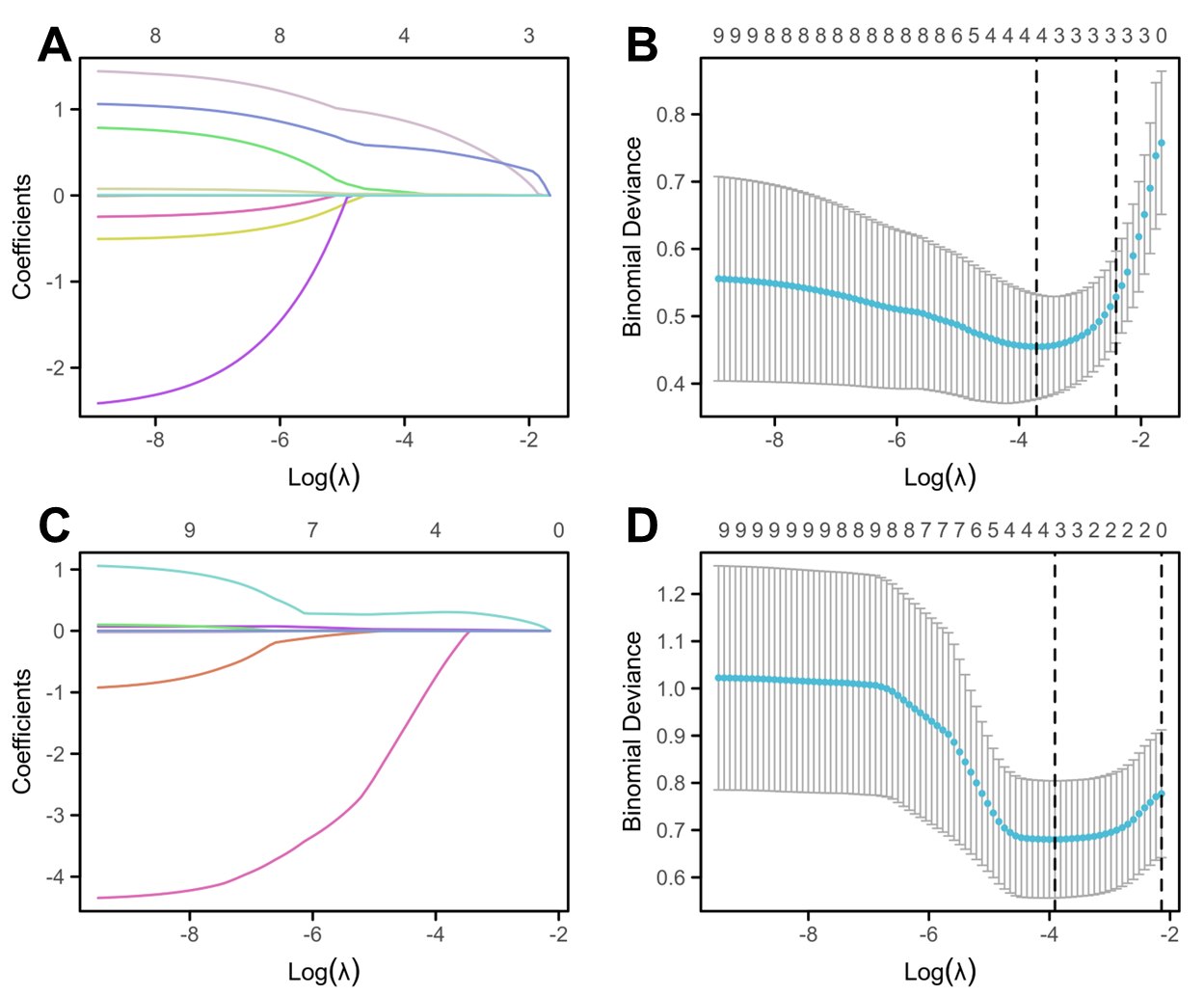


**Figure s1.** **Parameters selected using LASSO logistic regression.** (A and B) The clinical scoring systems were selected. (C and D) The inflammation-related markers were selected. The optimal parameter (λ) was selected using 10-fold cross-validation via 1 standard error of the minimum criteria.


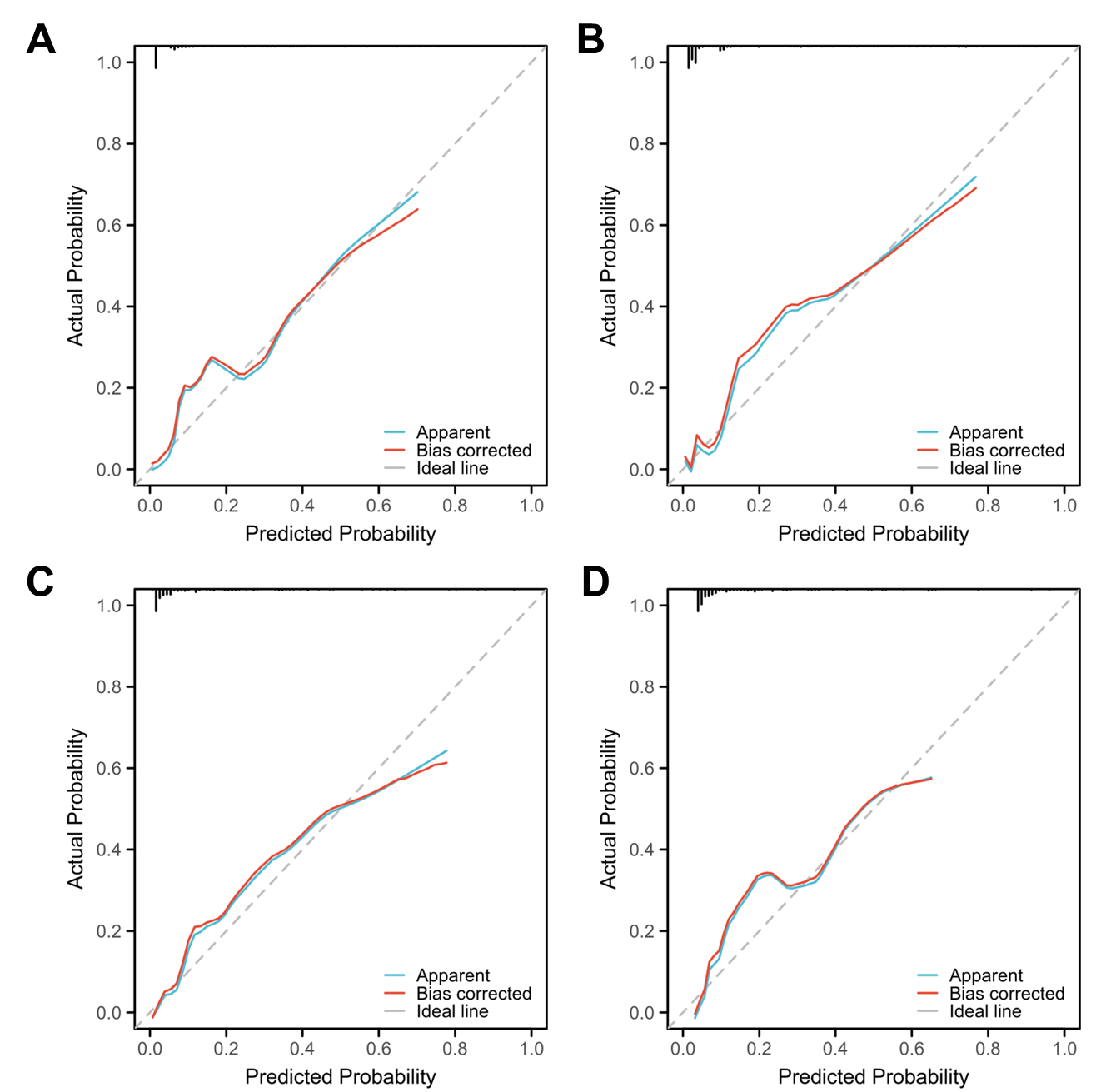


**Figure s2. Calibration curves of the nomograms for predicting 28-day mortality in COVID-19 patients.** A: Presepsin+qSOFA (p=0.81); B: Presepsin+CURB-65 (p=0.19); C: Presepsin+NEWS2 (p=0.75); D: Presepsin+CAR (0.33). Abbreviations: qSOFA, quick sequential organ failure assessment; NEWS2, National Early Warning Score 2; CAR, C-reactive protein-to-albumin ratio.


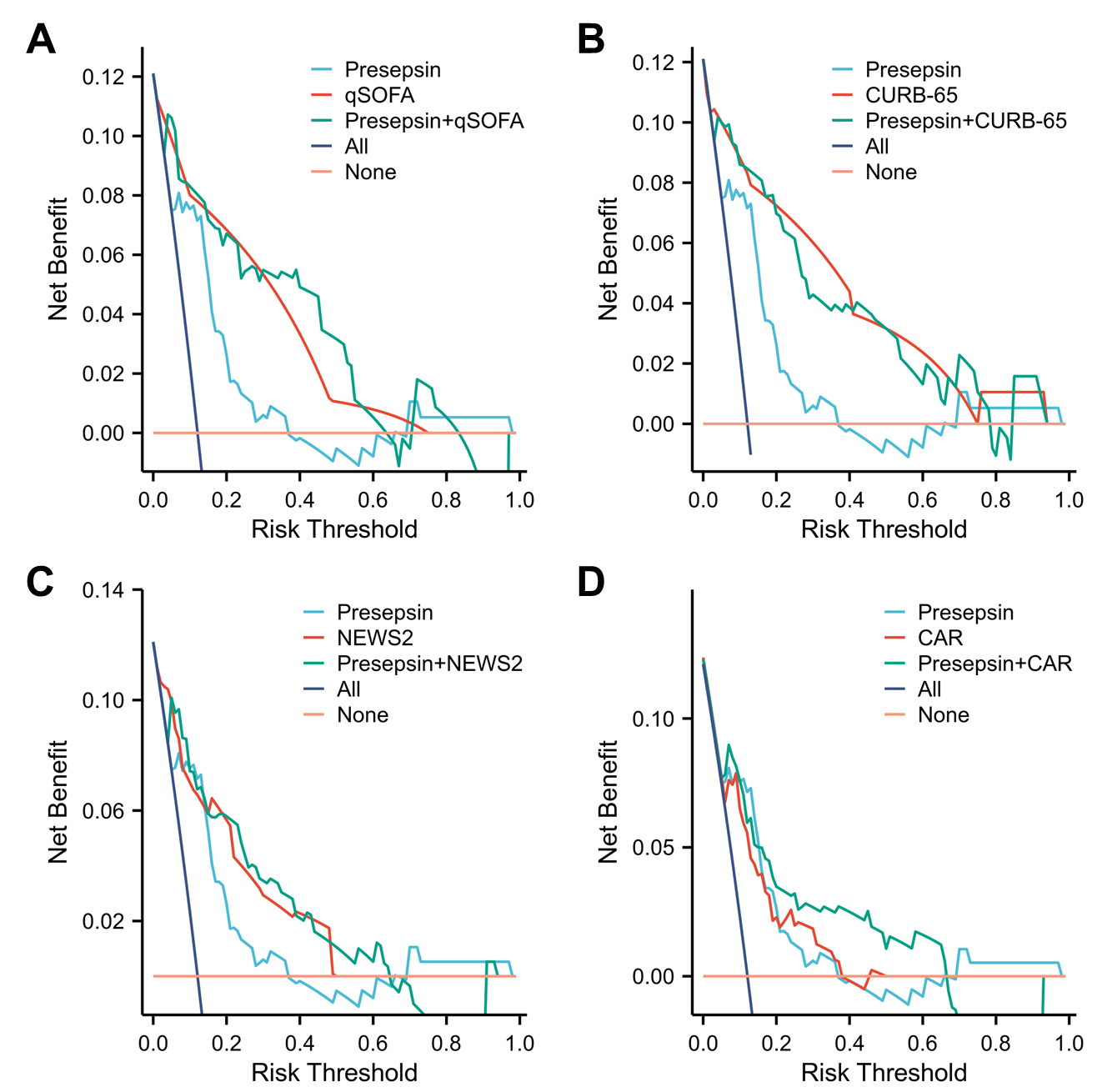


**Figure s3. Decision curve analysis of the nomograms for predicting 28-day mortality in COVID-19 patients.** A: Presepsin+qSOFA; B: Presepsin+CURB-65; C: Presepsin+NEWS2; D: Presepsin+CAR. Abbreviations: qSOFA, quick sequential organ failure assessment; NEWS2, National Early Warning Score 2; CAR, C-reactive protein-to-albumin ratio.
